# Dairy Consumption and Risk of Conventional and Serrated Precursors of Colorectal Cancer: A Systematic Review and Meta-Analysis of Observational Studies

**DOI:** 10.1155/2021/9948814

**Published:** 2021-05-26

**Authors:** Li-Liangzi Guo, Yu-Ting Li, Jun Yao, Li-Sheng Wang, Wei-Wei Chen, Kai-Yin He, Lin Xiao, Shao-Hui Tang

**Affiliations:** ^1^Department of Gastroenterology, The First Affiliated Hospital, Jinan University, Guangzhou, Guangdong 510632, China; ^2^Department of Gastroenterology, Shenzhen People's Hospital (The Second Clinical Medical College, Jinan University, The First Affiliated Hospital, Southern University of Science and Technology), Shenzhen, Guangdong 518020, China

## Abstract

**Objective:**

The consumption of dairy is associated with decreased risk of colorectal cancer (CRC), but few studies have assessed the relationship between dairy consumption and precursors of CRC. Therefore, we performed the first meta-analysis to further evaluate this association.

**Methods:**

PubMed, Embase, Scopus, and Web of Science databases were searched through July 2020 for observational studies. Study-specific risk estimates for the highest versus lowest category were pooled using the random-effects and fixed-effects model. The methodological quality of included studies was assessed using the ROBINS-I Scale.

**Results:**

A total of 12 studies were included (3 cohort studies and 9 case-control studies). Compared with the lowest level consumption, fermented dairy products had a decreased risk of precursors of CRC in both cohort (RR = 0.92 95% CI: 0.87–0.97) and case-control studies (RR = 0.98 95% CI: 0.96–0.99). Total dairy (RR = 0.80 95% CI: 0.68–0.96) and cheese (RR = 0.96 95% CI: 0.93–0.99) consumption was inversely associated with the risk in case-control studies whereas yogurt consumption was inversely associated with the risk in cohort studies (RR = 0.91 95%CI: 0.86–0.96). No significant associations were found for consumption of total milk and non/low-fat milk. For dose-response analyses, evidence of linear association was found in total dairy and yogurt consumption. The risk decreased by 12% for an increment of 200 g/d total dairy consumption (RR = 0.88 95% CI: 0.81–0.95) and decreased by 8% for an increment of 50 g/d yogurt consumption (RR = 0.92 95% CI: 0.85–0.99).

**Conclusions:**

Fermented dairy products, specifically yogurt and cheese, were significantly associated with decreased risk of conventional and serrated precursors of colorectal cancer.

## 1. Introduction

According to GLOBOCAN 2018, colorectal cancer (CRC) is the third most frequently diagnosed cancer and the second leading cause of cancer death. It is estimated that there will be over 1.8 million new CRC cases and 881,000 deaths in 2018 [1]. It is well known that cancer is a complicated disease caused by the interaction of environmental and genetic factors, such as smoking, alcohol, and diet [2]. Various dietary factors have been found to be important protective factors for CRC. For example, the Mediterranean diet might decrease the risk of CRC [3, 4]. In an umbrella review of observational studies, intake of total dairy was associated with decreased risk of cardiovascular disease, hypertension, and fatal stroke [5]. The latest report of World Cancer Research Fund (WCRF) and American Institute of Cancer Research (AICR) also reached strong evidence that dairy products intake can help protect against CRC [6].

Adenomas (adenomatous polyps, AP) and a subset of serrated lesions (traditional serrated adenoma (TSA) and sessile serrated adenoma/polyp (SSA/P) are two major subtypes of the precursors of CRC. A previous meta-analysis has shown an increased risk of colorectal adenoma (CRA) with the intake of red and processed meat [7, 8]; a decreased risk with increased intake of dietary fiber [9], calcium [10], magnesium [11], selenium, [12] and coffee [13]; and a null association with white meat [14]. However, the relationship between dairy consumption and precursors of CRC is still uncertain. To better understand this relationship, we combined all published epidemiologic studies on the association between dairy intake and precursors of CRC risk and then conducted a dose-response meta-analysis.

## 2. Methods

### 2.1. Design

The protocol of this meta-analysis was registered in PROSPERO (CRD42020192846). This study was reported according to the Preferred Reporting Items for Systematic Reviews and Meta-Analysis (PRISMA) guidelines [15]. The completed PRISMA checklist is provided online. PICO format (population, intervention, comparison, outcome) was employed to answer the research question: “Are dairy associated with the occurrence of conventional and serrated precursors of colorectal cancer.” Population: adults with conventional adenomas (including tubular adenoma, tubulovillous adenoma, villous adenoma, and adenoma with high-grade dysplasia) and serrated precursors of colorectal cancer (TSA and SSA/P); cases with hyperplastic polyps (HP) were excluded from the analyses. Intervention: total dairy product intake (total dairy, milk, cheese, butter, yogurt, or other dairy products). Comparison: adults without colorectal adenoma or serrated lesion. Outcome: the occurrence of conventional adenomas and serrated lesions.

### 2.2. Search Strategy

We searched PubMed, Embase, Scopus, and Web of Science database in English until April 2020. The following search terms were used: “dairy” or “dairy products” or “fermented milk products” or “cultured milk products” or “fermented dairy products” or subtypes of dairy products (i.e., “milk” or “yogurt” or “yoghurt” or “cheese” or “hard cheese” or “cottage cheese” or “cheddar” or “butter” or “buttermilk” or “cream” or “ice cream” or “Kefir”; NOT: “milk protein” or “whey protein”; AND: “colorectal adenoma” or “colorectal polyp” or “colorectal lesion” or “colorectal neoplasm” or “colorectal tumor” or “colorectal carcinoma” or “colorectal cancer” without restrictions. Titles and abstracts were screened independently by two reviewers (Liliangzi Guo and Yuting Li) to exclude irrelevant articles. Then, the full texts were retrieved to further increase the potentially relevant articles.

### 2.3. Study Selection

Inclusion criteria: (1) adult participants>18 years of age; (2) cohort studies, case-control studies, or cross-sectional studies that investigated the association between dairy consumption and risk of conventional adenomas and serrated lesions; (3) diagnosis: colorectal adenomas and serrated lesions that were determined by histology; (4) studies that reported the risk estimates (relative risk (RR), odds ratio (OR), or hazard ratio (HR)) with their corresponding 95% confidence interval (CI) or available original data allowing us to compute the 95% CI; (5) if the published studies reported data for specific subgroups, results for the whole population were considered in this meta-analysis; (6) if the original publications provided several independent studies, they were considered as separate studies in the following data analysis.

Exclusion criteria: (1) animal studies; (2) studies conducted on children, adolescents, or pregnancy women; (3) nonoriginal papers (reviews, editorials, or commentaries); (4) meta-analysis studies; (5) studies that did not provide enough data on dairy products consumption and risk estimates; (6) duplicate reports and abstracts; and (7) studies that investigated the risk of CRC or the recurrence of colorectal adenomas or serrated lesions.

### 2.4. Data Extraction and Quality Assessment

The data extracted from each study included the name of first author, publication year, study region, study design, sample size, type of colorectal lesion, dairy products categories, the risk estimates with their 95% CIs for each category of exposure variables and adjusted covariates in the multivariable analysis. We extracted the risk estimates that reflected the greatest degree of adjustment for potential confounders.

Dose-response analysis requires the distribution of cases and controls, person-years or noncases, and risk estimates with 95% CIs for at least three quantitative categories of exposure to dairy. The median or mean level of dairy intake for each category was assigned to the corresponding RR for every study. The interval size of the open-ended highest category was assumed as the closest interval while the lowest was considered as zero [9]. Two investigators (Liliangzi Guo and Weiwei Chen) independently extracted the data. Any disagreements were resolved by consulting the third investigator (Shaohui Tang).

Two investigators (Weiwei Chen and Kaiyin He) independently assessed the study quality based on the Risk of Bias in Nonrandomized Studies of Interventions (ROBINS-I) tool which contains seven domains. According to ROBINS-I guidance, the study judged to be at low risk in all seven domains was considered as low risk of bias; the study judged to be at low or moderate risk in all domains was considered as moderate risk of bias; the study judged to be at serious risk or critical risk in at least one domain was considered as serious risk of bias or critical risk of bias, respectively [16].

The definition of the dairy category (Table S1) in this meta-analysis is listed as follows. Total dairy includes total milk and fermented dairy product. Total milk includes non/low-fat milk and whole milk. The fermented dairy product includes yogurt, cheese, and cottage cheese.

### 2.5. Statistical Analysis

The results were expressed in terms of RR and 95% CI for the highest versus lowest category of dairy products consumption. The standard error of the logarithmic OR/RR of each study was calculated and taken as the estimated variance of the logarithmic OR/RR. The inverse variance method was adopted (DerSimonian and Laird, 1986) [17]. Dose-response analysis was also conducted and *P* < 0.05 was considered statistically significant. Cochran's *Q*-test and *I*^2^statistics were used to assess the heterogeneity of individual studies. *I*^2^ > 50% and *P* < 0.1 were considered as statistically significant heterogeneity [18]. Fixed-effects model and random-effects model were adopted as the pooling method. We used the random-effects model to calculate the pooled RRs and 95 CIs when the evidence of heterogeneity was present (>50%). Otherwise, if there was no obvious heterogeneity (<50%), the fixed-effects model was used. Subgroup analyses were performed to explore the source of heterogeneity in this study.

The subgroup analyses were performed according to study design, geographic location, patient sex, number of cases, size of adenoma, type of colorectal lesion, study quality, and type of food frequency questionnaire (FFQ) with dairy consumption if data were permitted. Sensitivity analyses were conducted to examine the stability of results by omitting one study at a time. Publication bias was assessed with Begg's test and Egger's test if ≥10 studies are available and it was considered to exist when *P* < 0.05. The trim and fill method was used to reduce the potential influence of publication bias [19].

Moreover, if ≥3 studies are available, a dose-response meta-analysis was carried out to estimate the trend between different exposure levels of dairy products and precursors of CRC using a random-effects meta-regression. Generalized least-squares trend (GLST) estimation modeling and spline curve modeling (MKspline STATA command) were used to estimate the dose-response relation analysis. Both linear and nonlinear dose-response analyses were performed with data from the included studies. The GLST and variance-weighted least-squares methods require median values for categories of intake levels. When medians and means were not presented, the category mid-point was used. If the dairy consumption was reported in servings/day, we converted it into grams/day as the following standard units: 200 g for total dairy, 200 g for milk (1 glass), 125 g for yogurt (1 cup), and 30 g for cheese [20]. If the cheese consumption was given in slices/day, we considered each slice as 25 g [21]. The dose-response results were presented for a 200, 200, 50, and 25 g/day increment for total dairy products, total milk, yogurt, and cheese, respectively. All statistical analyses were performed using STATA, version 12.0 (Stata Corp., College Station, TX, USA).

## 3. Results

### 3.1. Search Results and Study Characteristics

**Figure 1:**
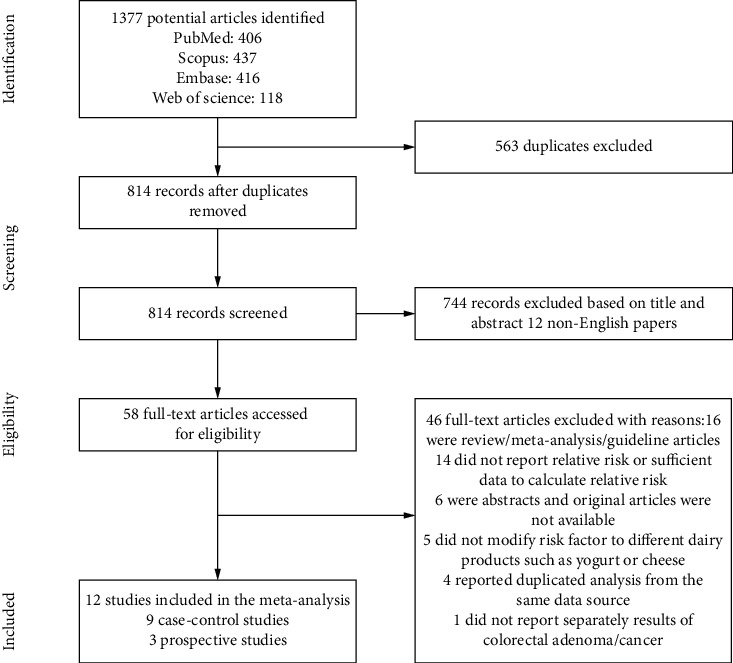
Flow of information through the different phases of the identification and selection of relevant studies examining the association between dairy consumption and the risk of the precursors of CRC.

Figure 1 shows the flow diagram of the identification and selection process of the included studies. Of the 1377 potentially relevant articles which initially retrieved, 563 duplicate articles were excluded and 58 articles remained for full-text review after screening the title and abstract. Among them, 46 articles were excluded (16 were review/meta-analysis/guideline articles, 14 did not report relative risk or sufficient data to calculate relative risk, 6 were abstracts and original articles were not available, 5 did not modify risk factor to different dairy products such as yogurt or cheese, 4 reported duplicated analysis from the same data source, and 1 did not report separately results of colorectal adenoma/cancer). In the end, a total of 12 eligible articles were included in this systematic review and meta-analysis: 9 case-control and 3 cohort studies.

The 12 included studies, published between 1991 and 2020, had 19957 cases with precursors of CRC. Of the studies, 6 were conducted in Europe, 4 in the United States, 1 in Iran, and 1 in Australia. Tables 1 and 2 show the main characteristics of the included studies.

### 3.2. Quality Assessment

All 12 studies were assessed to have a moderate risk of bias.  Table S2 showed the study quality and bias risk of each domain of the included studies.

### 3.3. Highest Consumption Compared with Lowest Consumption Analysis

As shown in Table 3, a total of 12 studies assessed the effect of dairy consumption on the likelihood of colorectal adenomas and serrated lesions, which include 3 cohort studies [22–24] (15124 cases) and 9 case-control study [25–33] (4833 cases).

#### 3.3.1. Total Dairy

**Figure 2:**
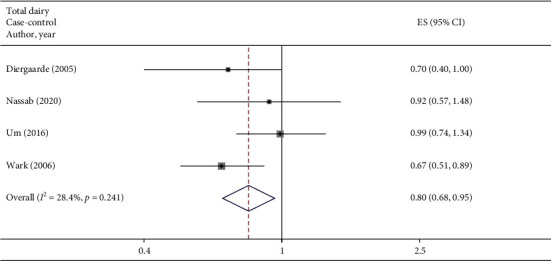
Fixed-effects meta-analysis of studies that examined total dairy consumption and risk of colorectal adenomas and serrated lesions. ES, effect size.

A total of 5 studies assessed the effect of total dairy consumption on the likelihood of colorectal adenomas and serrated lesions, which include 1 cohort study [23] (516 cases) and 4 case-control studies [26, 27, 32, 33] (1862 cases). A significantly negative association of total dairy intake with colorectal adenomas and serrated lesions was observed among overall studies (RR = 0.80 95% CI: 0.69–0.93, *P*=0.003; *I*^2^ = 4.6%) (Figure 2). Evidence in favor of the association was weaker among cohort studies (RR = 0.80 95% CI: 0.61–1.04, *P*=0.097) when compared with case-control studies (RR = 0.80 95% CI: 0.68–0.96, *P*=0.013; *I*^2^ = 28.4%).

#### 3.3.2. Total Milk

**Figure 3:**
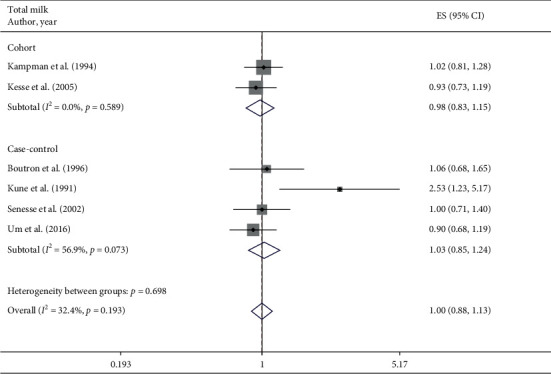
Fixed-effects meta-analysis of studies that examined total milk consumption and risk of colorectal adenomas and serrated lesions. ES, effect size.

A total of 6 studies assessed the effect of total milk consumption on the likelihood of colorectal adenomas and serrated lesions, which included 2 cohort studies [22, 23] (1197 cases) and 4 case-control studies [25, 29, 31, 32] (1560 cases). The pooled summary effect size indicated no significant association in overall (RR = 1.00 95% CI: 0.88–1.13, *P*=0.983; *I*^2^ = 32.4%), cohort (RR = 0.98 95% CI: 0.83–1.15, *P*=0.782; *I*^2^ = 0%), and case-control (RR = 1.03 95% CI: 0.85–1.24, *P*=0.983; *I*^2^ = 56.9%) studies (Figure 3).

#### 3.3.3. Non/Low-Fat Milk

A total of 3 studies assessed the effect of non/low-fat milk consumption on the likelihood of colorectal adenomas and serrated lesions, which include 1 cohort study [22] (681 cases) and 2 case-control studies [25, 32] (1149 cases). No significant association between non/low-fat milk intake with colorectal adenomas and serrated lesions was observed among overall studies (RR = 0.96 95% CI: 0.81–1.14, *P*=0.659; *I*^2^ = 0%), cohort studies (RR = 0.98 95% CI: 0.75–1.28, *P*=0.880), and case-control studies (RR = 0.95 95% CI: 0.75–1.19, *P*=0.649; *I*^2^ = 0%).

#### 3.3.4. Fermented Dairy Products

**Figure 4:**
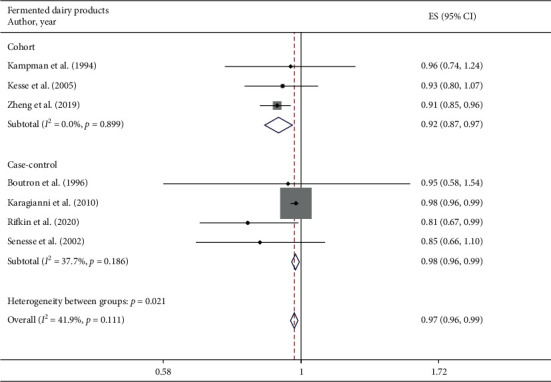
Fixed-effects meta-analysis of studies that examined fermented dairy products consumption and risk of colorectal adenomas and serrated lesions. ES, effect size.

A total of 7 studies assessed the effect of fermented dairy product consumption on the likelihood of colorectal adenomas and serrated lesions, which included 3 cohort studies [22–24] (15124 cases) and 4 case-control studies [25, 28, 30, 31] (2922 cases). A significantly negative association of fermented dairy products intake with colorectal adenomas and serrated lesions was observed among overall studies (RR = 0.97 95%CI: 0.96–0.99, *P* ≤ 0.001; *I*^2^ = 41.9%), cohort studies (RR = 0.92 95%CI: 0.87–0.97, *P*=0.002; *I*^2^ = 0%), and case-control studies (RR = 0.98 95% CI: 0.96–0.99, *P*=0.005; *I*^2^ = 37.7%) (Figure 4).

#### 3.3.5. Yogurt

**Figure 5:**
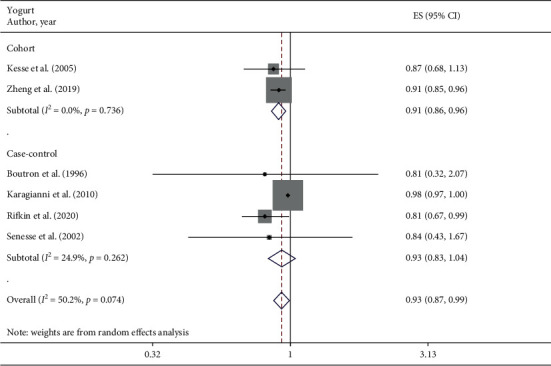
Fixed-effects meta-analysis of studies that examined yogurt consumption and risk of colorectal adenomas and serrated lesions. ES, effect size.

A total of 6 studies assessed the effect of yogurt consumption on the likelihood of colorectal adenomas and serrated lesions, which include 2 cohort studies [23, 24] (14443 cases) and 4 case-control studies [25, 28, 30, 31] (2922 cases). A negative association of yogurt intake with colorectal adenomas and serrated lesions was observed among overall studies (RR = 0.93 95% CI: 0.87–0.99, *P*=0.029; *I*^2^ = 50.2%). Evidence in favor of the association was weaker among case-control studies (RR = 0.93 95% CI: 0.83–1.04, *P*=0.218; *I*^2^ = 24.9%) when compared with cohort studies (RR = 0.91 95%CI: 0.86–0.96, *P* ≤ 0.001; *I*^2^ = 0%) (Figure 5).

#### 3.3.6. Cheese

**Figure 6:**
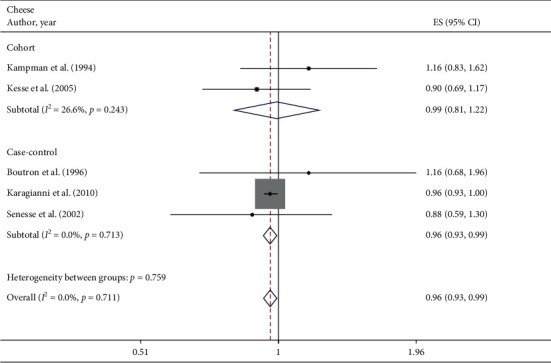
Fixed-effects meta-analysis of studies that examined cheese consumption and risk of colorectal adenomas and serrated lesions. ES, effect size.

A total of 5 studies assessed the effect of cheese consumption on the likelihood of colorectal adenomas and serrated lesions, which include 2 cohort studies [22, 23] (1197 cases) and 3 case-control studies [25, 28, 31] (776 cases). Cheese intake was negatively associated with colorectal adenomas and serrated lesions among overall studies (RR = 0.96 95% CI: 0.93–0.99, *P*=0.017; *I*^2^ = 0%) and case-control studies (RR = 0.96 95%CI: 0.93–0.99, *P*=0.016; *I*^2^ = 0%). However, the results from the cohort study showed no significant association (RR = 0.99 95%CI: 0.81–1.22, *P*=0.940; *I*^2^ = 26.6%) (Figure 6).

### 3.4. Dose-Response Meta-Analysis

**Figure 7:**
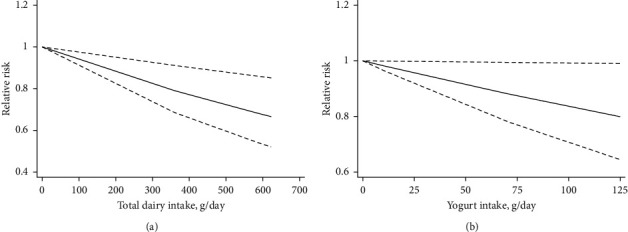
The Linear dose-response meta-analysis of total dairy (a) and yogurt (b) intake with relative risk of colorectal adenomas and serrated lesions. Weights are from the fixed-effects analysis. Solid line represents the linear trend. Lines with short dashes represent the 95% CI.

Both linear and nonlinear dose-response analyses were performed. The potential nonlinear association was examined using restricted cubic splines with 4 knots fixed at the 5^th^, 35^th^, 65^th^, and 95^th^ percentiles of the distribution. Combining data from 3 studies [26, 30, 33], trend meta-analysis showed a significant negative dose-response relationship in total dairy (*P*_−nonlinearity_ = 0.947) and yogurt (*P*_−nonlinearity_ = 0.794) consumption from linearity. We found that 200 g/d increment in total dairy consumption could decrease 12% risk of colorectal adenomas and serrated lesions using the fixed-effect model with no heterogeneity (RR = 0.88, 95%CI: 0.81–0.95, *P*=0.001; *P*_*h*_ = 0.658) (Figure 7(a)). Further, we found the risk of colorectal adenomas and serrated lesions decreased by 8% with an increment of 50 g yogurt using the fixed-effect model (RR = 0.92, 95% CI: 0.85–0.99, *P*=0.037; *P*_*h*_ = 0.367) (Figure 7(b)).

### 3.5. Subgroup and Sensitivity Analyses

The results of subgroup analyses conducted to explore the sources of heterogeneity were detailed shown in Table S3. Intake of total dairy, fermented dairy products, yogurt, and cheese was significantly associated with a decreased risk of colorectal adenomas and serrated lesions in most subgroup analysis. When stratified by sex, an inverse association was observed for intake of fermented dairy products in both men (SRR = 0.85, 95% CI: 0.77–0.93) and women (SRR = 0.92, 95% CI: 0.87–0.98). When stratified by the size of adenoma, an inverse association was observed for intake of yogurt and cheese in studies with a size of 10 mm or more (SRR = 0.71 95% CI: 0.51–0.99; SRR = 0.96 95% CI: 0.93–0.99) but not among studies with a size of less than 10 mm (SRR = 1.01 95% CI: 0.81–1.25; SRR = 1.08 95% CI: 0.68–1.71). Intake of total milk was not significantly associated with the risk of colorectal adenomas and serrated lesions in subgroup analyses. Too few studies of non/low-fat milk precluded any meaningful subgroup analysis.

Sensitivity analyses were conducted to evaluate the influence of a single study on the overall risk estimate by omitting one study in each turn (Table S4). In the analysis of total milk, the heterogeneity decreased from 56.9% to 0% when omitting the study by Kune et al. In the analysis of yogurt, the heterogeneity decreased from 50.2% to 0% when omitting the study by Karagianni et al.

## 4. Discussion

By pooling the 12 observational studies (9 case-control and 3 cohort studies) eventually included, this present meta-analysis was the first study that conclusively indicated an inverse association between fermented dairy products including yogurt and cheese and the risk of conventional adenomas and serrated lesions, of which fermented dairy products intake showed a decreased trend in both cohort and case-control studies but cheese and yogurt consumption display an inverse association only in case-control studies or cohort studies. No significant associations were found for the consumption of total milk and non/low-fat milk. Very few studies have evaluated the association between whole milk and cottage cheese with the risk of conventional adenomas and serrated lesions. The negative associations of total dairy, yogurt, and cheese were not supported by cohort studies which may be explained by many reasons such as the differences in study design, study quality, category of the intake frequency, amounts of dairy intake, and potential confounders.

CRC is arising through three major pathways, including adenoma-carcinoma sequence, serrated pathway, and inflammatory pathway [34]. It was estimated that approximately 10–15% of the sporadic CRC were progressed from a serrated polyp and 85–90% were progressed from adenomatous polyps. The subset of serrated polyps includes HP (hyperplastic polyp), TSA, and SSA/P [35]. HP is the most prevalent type of serrated polyps which is the lack of malignant potential [36]. Therefore, we excluded the data investigating risk between dairy and HP. No more than 10% of the adenomas will progress to CRC. Compared with nonadvanced adenomas, advanced adenomas (≥10 mm in diameter, villous histology, or high-grade dysplasia, with or without >3 adenomas) are more likely to develop into malignancy [35]. Because high-risk adenoma is more likely to develop into malignancy than low-risk adenoma, the data of high-risk adenoma are more informative for preventive strategies. The result of this meta-analysis showed a negative association between yogurt and cheese intake and adenoma ≥10 mm, suggesting that fermented dairy products may play a protective role against the progression of CRC. However, the result should be interpreted with caution because only a few studies were involved in this meta-analysis.

Dairy foods may decrease the risk of precursors of CRC through several mechanisms. Dairy foods are the main source of calcium in the diet. In the most recent report from the WCRF/AICR, low calcium intake may increase CRC risk. A previous observational study has shown a negative association between supplemental calcium intake and CRC risk [37]. On the one hand, calcium can decrease cell proliferation induced [38].

On the other hand, calcium can have an impact on several intracellular pathways leading to apoptosis in transformed cells and differentiation in normal cells [39, 40]. The results of RCT showed that, in normal appearing colorectal mucosa of individuals with a history of adenoma, the expression of APC (adenomatous polyposis coli) and *β*-catenin is modified by calcium supplementation [41, 42]. Moreover, besides calcium, vitamin D, and folate are also micronutrients which might have protective effects [43].

We found that total dairy and fermented dairy products were inversely associated with the risk of the precursors of CRC while total milk was not associated with the risk. There are several mechanisms proposed to explain how fermented dairy food decreases the risk of precursors of CRC. Since the process of fermentation, fermented dairy foods contain plentiful probiotic such as *Lactobacillus* and *Bifidobacterium* [44–46]. The microbiota in the human body can form a microenvironment to alter cancer susceptibility and progression [47]. In a case-control study, CRC-associated microbiota was found to change with the degree of malignancy along the adenoma-carcinoma sequence [48]. According to this evidence, the gut microbiome, which can correct microbiota composition, modulate the innate immune system, restore gut barrier function, prevent pathogen colonization, and exert selective cytotoxicity against tumor cells, plays an important role in the development of CRC [49]. Yogurt has been recommended by the Dietary Guideline for Americans [50]. Live microorganisms such as *Streptococcus thermophilus* and *Lactobacillus delbrueckii* subsp. *bulgaricus*, two lactic acid bacteria used to ferment, may prevent carcinogenesis from initiating [51]. Various studies also have reported a protective effect of probiotics or prebiotics in CRC mice models as reviewed [52]. Furthermore, yogurt has been suggested to induce gastrointestinal hormone secretion [53, 54].

Our systematic review and meta-analysis had several strengths. Most of the current meta-analysis focus on the relationship between dietary factors such as milk or dairy products and the risk of CRC. We conducted the first meta-analysis of the association between dairy consumption and the risk of precursors of CRC including the conventional and serrated lesions, based on highest versus lowest analysis, linear and nonlinear dose-response meta-analysis. We included both cohort and case-control studies through a systematic search. All of the included studies used a validated FFQ to assess dairy consumption. Further, a significantly inverse dose-response relationship was observed between total dairy and yogurt consumption and the risk of precursors of CRC, which may strengthen the reliability of the results of our hypothesis. We also carried out a sensitivity analysis to investigate whether a particular study could explain the results.

However, our meta-analysis had several limitations. The main limitation of the study was a small number of included studies and subjects, so further subgroup analyses were not able to perform according to anatomical location and histology type. Dose-response analyses of specific types of dairy products were also limited because a few studies reported the modifiable risk factor with 95% CI to different quantitative categories of exposure to dairy products. Secondly, the observed inverse association between dairy intake and precursors of CRC may be due to unmeasured or potential residual confounding, although the quality of 12 included studies was evaluated to have a moderate risk of bias by ROBINS-I tool. Although some known confounding factors were adjusted in most of the studies, not all potential confounders were adjusted for in every study. We found that the association between total dairy, fermented dairy products, yogurt, and cheese persisted in most subgroups, with adjustment for confounding factors. Thirdly, significant heterogeneity was observed among studies. However, there was no evidence of significant heterogeneity found between subgroups analyses. Fourthly, most of the case-control studies may not avoid recall and selection bias, especially dietary recall bias. Lastly, potential publication bias might have influenced the results.

## 5. Conclusion

This systematic review and meta-analysis was the first to assess the relationship between different dairy products and the precursor of CRC risk as well as the first to explore a linear association between them. In conclusion, our study suggests an inverse relationship between total dairy, fermented dairy products, yogurt, cheese, and risk of precursors of CRC, though the evidence was limited. However, no harmful effects were found between the intake of total milk and the risk of the precursor of CRC. Knowledge of risk factors associated with the precursors of CRC is important in prevention strategies. More large and precise prospective studies, as well as clinical trials, are needed to further investigate the associations and mechanisms between them. Future studies should also focus on the interactions between gut microbiota and environmental factors and their influences on colorectal carcinogenesis.

## Figures and Tables

**Table 1:** Characteristic of the 3 cohort studies included in the meta-analysis investigating the effect of dairy consumption on the risk of precursors of CRC.

Author/year/country	Study characteristics	Age and sex	Number of cases	Number of controls	Dietary assessments	Exposure	Contrast (highest vs. lowest)	OR/RR (95% CI)	Adjustments
Kampman et al./1994/USA	Undergone a sigmoidoscopy or colonoscopy within the follow-up period (HPFS, 1986–1990: 9490 men; NHS, 1980–1988: 8925 women)	HPFS: 40–75 years, men NHS: 30–55 years, women	331 men and 350 women with adenomatous polyps of the left colon or rectum	9159 men and 8585 women with endoscopic findings negative for adenoma	Validated FFQ	Milk (whole)	C5 vs. C1		Age, total energy, family history, and saturated fat intake
							HPFS	0.75 (0.29–1.91)	
							NHS	1.35 (0.76–2.41)	
						Milk (skim/lowfat)	C5 cs C1		
							HPFS	1.06 (0.72–1.54)	
							NHS	0.91 (0.63–1.30)	
						Fermented dairy products	C5 vs. C1		
							HPFS	1.06 (0.72–1.57)	
							NHS	0.89 (0.63–1.25)	
						Hard cheese	C4 vs. C1		
							HPFS	1.28 (0.88–1.86)	
							C5 vs. C1		
							NHS	0.81 (0.40–1.67)	

Kesse et al./2005/France	E3N-EPIC: 1933 women who had reported diagnosis of a colorectal polyp between the return of the dietary questionnaire (10993–1995) and the endpoint of the analysis (December 1997)	40–65 years, women	516 women with adenoma	4804 polyp-free women	Validated FFQ-208	Total dairy products	>424.29 g/d vs. <184.83 g/d	0.80 (0.62–1.05)	Educational lever, current smoking status, family history of colon cancer, body mass index, physical activity level and energy and alcohol intake
						Milk		0.93 (0.73–1.19)	
						Yogurt		0.87 (0.68–1.13)	
						Cottage cheese		1.01 (0.80–1.29)	
						Cheese		0.90 (0.69–1.17)	

Zheng et al./2020/USA	HPFS: 32606 men NHS: 55743 women undergone lower endoscopy between 1986 and 2012.	NA, M + F	5811 adenomas in men and 8116 adenomas in women	26795 men and 47627 women with endoscopic findings negative for adenoma	Validated FFQ	Yogurt	HPFS≧2/week vs. never	Conventional adenomas 0.81 (0.71–0.94)	Age, time period of endoscopy, number of reported endoscopies, time since most recent endoscopy and reason for current endoscopy, height, body mass index, family history of CRC, diabetes, pack-years of smoking, alcohol intake, physical activity in METs, regular use of aspirin, regular NSAIDs use, total vitamin D intake, nonyogurt dairy intake, total calorie intake, red and processed meat intake, dietary fiber intake, total folate intake, alternative healthy eating Index-2010, total calcium intake, menopausal status, and menopausal hormone use.
								Serrated lesion 0.89 (0.74–1.07)	
								Conventional adenomas and serrated lesions 0.78 (0.59–1.04)	
							NHS ≧2/week vs. never	Conventional adenomas 0.98 (0.88–1.09)	
								Serrated lesion 0.92 (0.82–1.04)	
								Conventional adenomas and serrated lesions 0.94 (0.76–1.17)	

HPFS: Health Professionals Follow-Up Study; NHS: Nurses' Health Study; FFQ: Food Frequency Questionnaire; RR: relative risk; OR: odds ratio; NA: not available; C5 vs. C1: frequency of consumption of one 8-ounce glass; C1, almost never; C5, more than once per day.

**Table 2:** Characteristic of the 9 case-control studies included in the meta-analysis investigating the effect of dairy consumption on the risk of precursors of CRC.

Author/year/country	Age and sex	Number of cases	Number of controls	Dietary assessments	Exposure	Contrast (highest vs. lowest)	OR (95% CI)	Adjustments
Boutron et al./1996/French	Aged 30–75, M + F	154 small adenomas (<10 mm)	426 polyp-free	Validated FFQ	Total milk	Q5 vs. Q1	1.1 (0.6–1.9)	Age, sex, and caloric intake
					Low-fat milk		1.0 (0.5–1.7)	
					Hard and semihard cheese		1.2 (0.6–2.4)	
					Cottage cheese	Q3 vs. Q1	1.0 (0.7–1.6)	
					Yogurt		1.3 (0.8–2.2)	
		208 large adenomas (>10 mm)	154 small adenomas (<10 mm)		Total milk	Q5 vs. Q1	1.0 (0.5–2.0)	
					Low-fat milk		1.1 (0.5–2.1)	
					Hard and semihard cheese		1.1 (0.5–2.5)	
					Cottage cheese	Q3 vs. Q1	1.1 (0.7–1.8)	
					Yogurt		0.5 (0.3–0.9)	

Diergaarde et al./2005/Dutch	Aged 18–75, M + F	278 CRA	414 polyp-free	Validated FFQ-178	Dairy products	≧495.0 g/d vs. ≦238.9 g/d	0.7 (0.4–1.0)	Age, gender, and total energy intake

Nasab et al./2020/Iran	Cases: aged 56.46 ± 10.01, controls: aged 55.08 ± 9.45, M + F	139 CRA	240 hospital control	Validated FFQ-148	Dairy	High vs. low	0.92 (0.57–1.48)	Energy, smoking, physical activity, age, calcium supplementation, history of diabetes and hypertension, cooking type, levels of salt intake, family history of cancer

Karagianni et al./2010/Greece	Cases: aged 30–77, controls: 33–80, M + F	52 advanced colorectal polyps	52 healthy control	Validated FFQ	Milk	Logistic regression	NA	Age, sex, smoking, physical activity, BMI, waist circumference, hypercholesterolemia, alcohol, yogurt, cheese, red meat, fish, fruits, vegetables, and garlic
					Yogurt		0.98 (0.97–1.00)	
					Cheese		0.96 (0.93–1.00)	

Kune et al./1991/Australia	Cases: aged 68 ± 9, controls: aged 65 ± 11, M + F	49 colorectal adenomatous polyps larger than 1 cm	727 community controls	Validated FFQ	Milk drinks	Logistic regression		Age, sex, vegetable, cruciferous vegetables, vitamin C, beef, pork, fish, fat, milk drinks, vitamin supplement, beer, family history of CRC in near relatives
						Male	3.70 (1.44–9.52)	
						Female	1.50 (0.50–4.54)	

Rifkin et al./2020/USA	TCPS: aged 40–75, JHBS: aged 40–85, M + F	Colorectal polyps TCPS: 181 SSP, 1536 AP JHBS: 96 SSP, 333 AP	Polyp-free controls TCPS: 3258 JHBS: 579	Validated FFQ-108	Yogurt	TCPS		TCPS: sex, study location, age, regular alcohol drinking status, BMI, smoking status, physical activity in the past 10 years, educational attainment, NSAID use, red meat intake, dietary energy intake, and frequency of nonyogurt dairy intake HBS: sex, age, cigarette use, overweight, prior colon polyp, history of cholecystectomy, diabetes mellitus diagnosis, hypertension diagnosis, hyperlipidemia diagnosis, physical activity and >10 alcohol drinks/week.
						Daily vs. never	AP 0.93 (0.69–1.25)	
							SSP 0.49 (0.19–1.24)	
						≧0.20 cups vs. none/rarely	AP 0.98 (0.79–1.22)	
							SSP 0.77 (0.43–1.36)	
						JHBS		
						1 or more/week vs. never	AP 0.75 (0.54–1.04)	
							SSP 0.76 (0.44–1.29)	

Senesse et al./2009/France	Aged 30–79, M + F	154 small adenomas (<10 mm), 208 large adenomas (≥10 mm)	427 polyp-free controls	Validated FFQ	Milk	Q4 vs. Q1	<10 mm 1.0 (0.6–1.7)	Age, gender, energy intake, body mass index, alcohol, and tobacco
							≧10 mm 1.0(0.7–1.7)	
					Yogurt	Q3 vs. Q1	<10 mm 1.2 (0.8–2.1)	
							≧10 mm 0.6 (0.4–1.0)	
					Cheese	Q4 vs. Q1	<10 mm 1.0 (0.5–1.7)	
							≧10 mm 0.8(0.5–1.4)	

Um et al./2016/USA	Aged 35–74, M + F	787 CRA	2033 polyp-free controls	Validated FFQ	Total milk products	Q5 vs. Q1	0.99 (0.74–1.34)	Study, age, sex, oxidative balance score, family history of colorectal cancer in first-degree relative, regular use of aspirin or nonsteroidal anti-inflammatory drugs, total energy intake, total fat intake (energy adjusted), supplemental calcium intake.
					Total milk	Q5 vs. Q1	0.90 (0.68–1.19)	
					Whole milk	Q2 vs. Q1	1.15 (0.89–1.49)	
					Nonfat milk	Q5 vs. Q1	0.92 (0.70–1.19)	

Wark et al./2006/Netherlands	Aged 18–75, M + F	658 CRA (81 K-ras^+^, 453 K-Ras^−^, 124 NA)	709 polyp-free controls	Validated FFQ-178	Dairy products	>238.2–474.6 vs. ≦238.2	0.89 (0.67, 1.18)	Sex, age, and total energy.
						>474.6 vs. >238.2–474.6	0.91 (0.68, 1.22)	

TCPS: Tennessee Colorectal Polyp Study; JHBS: Johns Hopkins Biofilm Study; FFQ: Food Frequency Questionnaire; AP: adenomatous polyp; SSP: sessile serrated polyp; OR: odds ratio; NA: not available; Q: quintiles.

**Table 3:** Analysis of highest versus lowest dairy consumption and risk of colorectal adenomas and serrated lesions.

	Factors	Number of studies	Pooled RR (95% CI)	*P* value	Heterogeneity	
					*I*^2^ (%)	*p*_*h*_
Total dairy	Total	5	0.80 (0.69, 0.93)	0.003	4.6	0.381
	Cohort	1	0.80 (0.61, 1.04)	0.097	—	—
	Case-control	4	0.80 (0.68, 0.96)	0.013	28.4	0.241

Total milk	Total	6	1.00 (0.88, 1.13)	0.983	32.4	0.193
	Cohort	2	0.98 (0.83, 1.15)	0.782	0	0.589
	Case-control	4	1.03 (0.85, 1.24)	0.983	56.9	0.073

Non/low-fat milk	Total	3	0.96 (0.81, 1.14)	0.659	0	0.890
	Cohort	1	0.98 (0.75, 1.28)	0.880	—	—
	Case-control	2	0.95 (0.75, 1.19)	0.649	0	0.656

Fermented dairy products	Total	7	0.97 (0.96, 0.99)	≤0.001	41.9	0.111
	Cohort	3	0.92 (0.87, 0.97)	0.002	0	0.899
	Case-control	4	0.98 (0.96, 0.99)	0.005	37.7	0.186

Yogurt	Total	6	0.93 (0.87, 0.99)	0.029	50.2	0.074
	Cohort	2	0.91 (0.86, 0.96)	≤0.001	0	0.736
	Case-control	4	0.93 (0.83, 1.04)	0.218	24.9	0.262

Cheese	Total	5	0.96 (0.93, 0.99)	0.017	0	0.711
	Cohort	2	0.99 (0.81, 1.22)	0.940	26.6	0.243
	Case-control	3	0.96 (0.93, 0.99)	0.016	0	0.713

*P*_*h:*_*P* value for heterogeneity.

## Data Availability

No data were used to support this study.

## Supplementary Materials

Supplementary Materials Supplemental Table 1. Definition of dairy products as described in the paper of 12 studies included in the meta-analysis. Supplementary Table 2. Bias risk of each domain of included studies assessed by ROBINS-I. Supplemental Table 3. Subgroup analyses of total dairy, fermented dairy products, total milk, yogurt, and cheese and the risk of colorectal adenomas and serrated lesions. Supplemental Table 4. The sensitivity analysis of the relationship between total milk intake and risk of colorectal adenomas and serrated lesions
