# Supplementary material for: Dairy Consumption and Risk of Conventional and Serrated Precursors of Colorectal Cancer: A Systematic Review and Meta-Analysis of Observational Studies
Source: J Oncol. 2021 May 26;2021:9948814. doi: 10.1155/2021/9948814 (PMC8172303; doi:10.1155/2021/9948814)
Supplement: Supplementary Materials — Supplemental Table 1. Definition of dairy products as described in the paper of 12 studies included in the meta-analysis. Supplementary Table 2. Bias risk of each domain of included studies assessed by ROBINS-I. Supplemental Table 3. Subgroup analyses of total dairy, fermented dairy products, total milk, yogurt, and cheese and the risk of colorectal adenomas and serrated lesions. Supplemental Table 4. The sensitivity analysis of the relationship between total milk intake and risk of colorectal adenomas and serrated lesions [file 9948814.f1.doc]

**Supplemental Table 1.** Definition of dairy products as described in the paper of 12 studies included in the meta-analysis.

| Exposure category in original paper | Exposure category in meta-analysis | Definition (if available) |
| --- | --- | --- |
| *Kampman et al, 1994* [21] |  |  |
| Milk | Total milk | Whole milk, skim/low fat milk |
| Fermented dairy products | Fermented dairy products | Yogurt, sour cream, cottage cheese, cream cheese, and other cheese |
| Hard cheese | Cheese | Not further defined |
| *Kesse et al, 2005* [22] |  |  |
| Total dairy | Total dairy | Not further defined |
| Milk | Total milk | Not further defined |
| Yogurt | Yogurt | Not further defined |
| Cottage cheese | Cottage cheese | Not further defined |
| Cheese | Cheese | Not further defined |
| *Zheng et al, 2019* [23] |  |  |
| Yogurt | Yogurt | Not further defined |
| *Boutron et al,* 1996 [24] |  |  |
| Milk(total) | Total milk | Not further defined |
| Low-fat milk | Non/low-fat milk | Not further defined |
| Cheese | Cheese | Not further defined |
| Cottage cheese | Cottage cheese | Not further defined |
| Yogurt | Yogurt | Not further defined |
| *Diergaarde et al, 2005* [25] |  |  |
| Dairy products | Total dairy | Not further defined |
| *Nassab et al, 2020* [26] |  |  |
| Dairy | Total dairy | Not further defined |

**Supplemental Table 1.** (Continued)

| Exposure category in original paper | Exposure category  in meta-analysis | Definition (if available) |
| --- | --- | --- |
| *Karagianni et al, 2010* [27] |  |  |
| Milk | Total milk | Not further defined |
| Yoghurt | Yogurt | Not further defined |
| Cheese | Cheese | Not further defined |
| *Kune et al, 1991* [28] |  |  |
| Milk drinks | Total milk | Not further defined |
| *Rifkin et al, 2020* [29] |  |  |
| Yogurt | Yogurt | Not further defined |
| *Senesse et al, 2002* [30] |  |  |
| Milk | Total milk | Not further defined |
| Yogurt | Yogurt | Not further defined |
| Cheese | Cheese | Not further defined |
| *Um et al, 2017* [31] |  |  |
| Total milk products | Total dairy | Milk, creams, ice cream and sherbet, fermented dairy products, cheeses, and butter. |
| Milk | Total milk | Whole milk, non/low-fat milk |
| Whole milk | Whole milk | Not further defined |
| Nonfat milk | Non/low-fat milk | Not further defined |
| *Wark et al, 2016* [32] |  |  |
| Dairy products | Total dairy | Dairy products, excluding cheese and butter |

**Supplementary Table 2.** Bias risk of each domain of included studies assessed by ROBINS-I.

| Study | Bias due to confounding | Bias in selection of participants | Bias in measurement of interventions | Bias due to departures from intended interventions | Bias due to missing data | Bias in measurement of outcomes | Bias in selection of the reported result | Overall bias |
| --- | --- | --- | --- | --- | --- | --- | --- | --- |
| Kampman et al, 1994 | Moderate risk | Low risk | Low risk | Low risk | Low risk | Low risk | Low risk | Moderate risk |
| Kesse et al, 2005 | Moderate risk | Low risk | Moderate risk | Low risk | Moderate risk | Low risk | Low risk | Moderate risk |
| Zheng et al, 2019 | Moderate risk | Low risk | Low risk | Low risk | Moderate risk | Low risk | Low risk | Moderate risk |
| Boutron et al, 1996 | Moderate risk | Low risk | Moderate risk | Low risk | Moderate risk | Low risk | Low risk | Moderate risk |
| Diergaarde et al, 2005 | Moderate risk | Low risk | Moderate risk | Low risk | Low risk | Low risk | Low risk | Moderate risk |
| Nassab et al, 2020 | Moderate risk | Low risk | Moderate risk | Low risk | Low risk | Low risk | Low risk | Moderate risk |
| Karagianni et al, 2010 | Moderate risk | Low risk | Moderate risk | Low risk | Low risk | Low risk | Low risk | Moderate risk |
| Kune et al, 1991 | Moderate risk | Low risk | Moderate risk | Low risk | Low risk | Low risk | Low risk | Moderate risk |
| Rifkin et al, 2020 | Moderate risk | Low risk | Moderate risk | Low risk | Moderate risk | Low risk | Low risk | Moderate risk |

**Supplemental Table 2.** (Continued)

| Study | Bias due to confounding | Bias in selection of participants | Bias in measurement of interventions | Bias due to departures from intended interventions | Bias due to missing data | Bias in measurement of outcomes | Bias in selection of the reported result | Overall bias |
| --- | --- | --- | --- | --- | --- | --- | --- | --- |
| Senesse et al, 2002 | Moderate risk | Low risk | Moderate risk | Low risk | Low risk | Low risk | Low risk | Moderate risk |
| Um et al, 2017 | Moderate risk | Low risk | Moderate risk | Low risk | Low risk | Low risk | Low risk | Moderate risk |
| Wark et al, 2016 | Moderate risk | Low risk | Moderate risk | Low risk | Moderate risk | Low risk | Low risk | Moderate risk |

**Supplemental Table 3.** Subgroup analyses of total dairy, fermented dairy products, total milk, yogurt and cheese and the risk of colorectal adenomas and serrated lesions.

|  | Fermented dairy products | | | | | Total dairy | | | | |
| --- | --- | --- | --- | --- | --- | --- | --- | --- | --- | --- |
| Subgroup | Number  of studies | Pooled RR (95%CI) | *P* value | Heterogeneity | | Number  of studies | Pooled RR (95%CI) | *P* value | Heterogeneity | |
|  |  |  |  | *I^2^* | *P_h_* |  |  |  | *I^2^* | *P_h_* |
| All studies | 7 | 0.97(0.96,0.99) | 0.000 | 41.9% | 0.000 | 5 | 0.80(0.69,0.93) | 0.003 | 4.6% | 0.381 |
| Design |  |  |  |  |  |  |  |  |  |  |
| Case-control | 4 | 0.98(0.96,0.99) | 0.005 | 37.7% | 0.186 | 4 | 0.80(0.68,0.96) | 0.013 | 28.4% | 0.241 |
| Cohort | 3 | 0.92(0.87,0.97) | 0.002 | 0% | 0.899 | 1 | 0.80(0.61,1.04) | 0.097 |  |  |
| Geographic location |  |  |  |  |  |  |  |  |  |  |
| Europe | 4 | 0.98(0.96,0.99) | 0.006 | 0% | 0.640 | 3 | 0.73(0.61,0.87) | 0.000 | 0% | 0.650 |
| United States | 3 | 0.90(0.85,0.96) | 0.000 | 0% | 0.480 | 1 | 0.99(0.74,1.33) | 0.947 | - | - |
| Others | 0 | - | - | - | - | 1 | 0.92(0.57,1.48) | 0.732 | - | - |
| Sex |  |  |  |  |  |  |  |  |  |  |
| Men | 4 | 0.85(0.77,0.93) | 0.000 | 0% | 0.557 | 0 | - | - | - | - |
| Women | 5 | 0.92(0.87,0.98) | 0.008 | 7.2% | 0.366 | 0 | - | - | - | - |
| Size of adenoma |  |  |  |  |  |  |  |  |  |  |
| Small (<10mm) | 3 | 0.98(0.85,1.13) | 0.786 | 19.2% | 0.004 | 0 | - | - | - | - |
| Large (≥10mm) | 4 | 0.80(0.64,1.02) | 0.067 | 77.9% | 0.290 | 0 | - | - | - | - |
| No. of cases |  |  |  |  |  |  |  |  |  |  |
| <280 | 1 | 0.98(0.97,1.00) | 0.010 | - | - | 1 | 0.92(0.57,1.48) | 0.732 | - | - |
| ≥280 | 6 | 0.90(0.86,0.95) | 0.000 | 0% | 0.866 | 4 | 0.79(0.68,0.92) | 0.002 | 21.9% | 0.279 |
| Adjustment for confounders |  |  |  |  |  |  |  |  |  |  |

**Supplemental Table 3.** (Continued)

|  | Fermented dairy products | | | | | Total dairy | | | | |
| --- | --- | --- | --- | --- | --- | --- | --- | --- | --- | --- |
| Subgroup | Number  of studies | Pooled RR (95%CI) | *P* value | Heterogeneity | | Number  of studies | Pooled RR (95%CI) | *P* value | Heterogeneity | |
|  |  |  |  | *I^2^* | *P_h_* |  |  |  | *I^2^* | *P_h_* |
| BMI |  |  |  |  |  |  |  |  |  |  |
| Yes | 5 | 0.97(0.96,0.99) | 0.000 | 61.2% | 0.036 | 1 | 0.80(0.61,1.04) | 0.097 | - | - |
| No | 2 | 0.96(0.76,1.20) | 0.711 | 0% | 0.970 | 4 | 0.80(0.68,0.95) | 0.013 | 28.4% | 0.241 |
| Smoking |  |  |  |  |  |  |  |  |  |  |
| Yes | 5 | 0.97(0.96,0.99) | 0.000 | 61.2% | 0.036 | 2 | 0.83(0.66,1.04) | 0.105 | 0% | 0.615 |
| No | 2 | 0.96(0.76,1.20) | 0.711 | 0% | 0.970 | 3 | 0.79(0.65,0.95) | 0.011 | 47.8% | 0.147 |
| Alcohol |  |  |  |  |  |  |  |  |  |  |
| Yes | 5 | 0.97(0.96,0.99) | 0.000 | 61.2% | 0.036 | 1 | 0.80(0.61,1.04) | 0.097 | - | - |
| No | 2 | 0.96(0.76,1.20) | 0.711 | 0% | 0.970 | 4 | 0.80(0.68,0.95) | 0.013 | 28.4% | 0.241 |
| Dietary energy intake |  |  |  |  |  |  |  |  |  |  |
| Yes | 6 | 0.90(0.86,0.95) | 0.000 | 0% | 0.866 | 5 | 0.80(0.69,0.93) | 0.003 | 4.6% | 0.381 |
| No | 1 | 0.98(0.97,1.00) | 0.010 | - | - | 0 | - | - | - | - |
| Physical activity |  |  |  |  |  |  |  |  |  |  |
| Yes | 4 | 0.97(0.96,0.99) | 0.001 | 67.5% | 0.027 | 2 | 0.83(0.66,1.04) | 0.105 | 0% | 0.615 |
| No | 3 | 0.91(0.77,1.08) | 0.268 | 0% | 0.791 | 3 | 0.79(0.65,0.95) | 0.011 | 47.8% | 0.147 |
| Folate |  |  |  |  |  |  |  |  |  |  |
| Yes | 1 | 0.91(0.86,0.97) | 0.002 | - | - | 0 | - | - | - | - |
| No | 6 | 0.98(0.96,0.99) | 0.004 | 5.7% | 0.380 | 5 | 0.80(0.69,0.93) | 0.003 | 4.6% | 0.381 |

***Supplemental Table 3.*** *(Continued)*

|  | Total milk | | | | |
| --- | --- | --- | --- | --- | --- |
| Subgroup | Number  of studies | Pooled RR (95%CI) | *P* value | Heterogeneity | |
|  |  |  |  | *I^2^* | *P_h_* |
| All studies | 6 | 1.00(0.88,1.13) | 0.983 | 32.4% | 0.193 |
| Design |  |  |  |  |  |
| Case-control | 4 | 1.03(0.85,1.24) | 0.983 | 56.9% | 0.073 |
| Cohort | 2 | 0.98(0.83,1.15) | 0.782 | 0% | 0.589 |
| Geographic location |  |  |  |  |  |
| Europe | 3 | 0.97(0.81,1.16) | 0.744 | 0% | 0.861 |
| United States | 2 | 0.97(0.81,1.16) | 0.737 | 0% | 0.497 |
| Others | 1 | 2.53(1.23,5.19) | 0.011 | - | - |
| Sex |  |  |  |  |  |
| Men | 2 | 1.79(0.51,6.33) | 0.366 | 83.4% | 0.012 |
| Women | 3 | 0.98(0.81,1.18) | 0.805 | 0% | 0.666 |
| Size of adenoma |  |  |  |  |  |
| Small (<10mm) | 2 | 1.04(0.71,1.54) | 0.828 | 0% | 0.810 |
| Large (≥10mm) | 2 | 1.00(0.69,1.45) | 1.000 | 0% | 1.000 |
| No. of cases |  |  |  |  |  |
| <280 | 1 | 2.53(1.23,5.19) | 0.011 | - | - |
| ≥280 | 5 | 0.97(0.85,1.10) | 0.640 | 0% | 0.944 |
| Adjustment for confounders |  |  |  |  |  |

***Supplemental Table 3.*** *(Continued)*

|  | Total milk | | | | |
| --- | --- | --- | --- | --- | --- |
| Subgroup | Number  of studies | Pooled RR (95%CI) | *P* value | Heterogeneity | |
|  |  |  |  | *I^2^* | *P_h_* |
| BMI |  |  |  |  |  |
| Yes | 2 | 0.95(0.78,1.16) | 0.723 | 0% | 0.734 |
| No | 4 | 1.03(0.88,1.21) | 0.637 | 56.7% | 0.074 |
| Smoking |  |  |  |  |  |
| Yes | 2 | 0.95(0.78,1.16) | 0.723 | 0% | 0.734 |
| No | 4 | 1.03(0.88,1.21) | 0.637 | 56.7% | 0.074 |
| Alcohol |  |  |  |  |  |
| Yes | 2 | 0.95(0.78,1.16) | 0.723 | 0% | 0.734 |
| No | 4 | 1.03(0.88,1.21) | 0.637 | 56.7% | 0.074 |
| Dietary energy intake |  |  |  |  |  |
| Yes | 5 | 0.97(0.85,1.10) | 0.640 | 0% | 0.944 |
| No | 1 | 2.53(1.23,5.19) | 0.011 | - | - |
| Physical activity |  |  |  |  |  |
| Yes | 1 | 0.93(0.73,1.19) | 0.560 | - | - |
| No | 5 | 1.02(0.89,1.18) | 0.748 | 42.5% | 0.138 |
| Folate |  |  |  |  |  |
| Yes | 0 | - | - | - | - |
| No | 6 | 1.00(0.88,1.13) | 0.983 | 32.4% | 0.193 |

**Supplemental Table 3.** (Continued)

|  | Yogurt | | | | | Cheese | | | | |
| --- | --- | --- | --- | --- | --- | --- | --- | --- | --- | --- |
| Subgroup | Number  of studies | Pooled RR (95%CI) | *P* value | Heterogeneity | | Number  of studies | Pooled RR (95%CI) | *P* value | Heterogeneity | |
|  |  |  |  | *I^2^* | *P_h_* |  |  |  | *I^2^* | *P_h_* |
| All studies | 6 | 0.93(0.87,0.99) | 0.029 | 50.2% | 0.074 | 5 | 0.96(0.93,0.99) | 0.017 | 0% | 0.711 |
| Design |  |  |  |  |  |  |  |  |  |  |
| Case-control | 4 | 0.93(0.83,1.04) | 0.218 | 24.9% | 0.262 | 3 | 0.96(0.93,0.99) | 0.016 | 0% | 0.713 |
| Cohort | 2 | 0.91(0.86,0.96) | 0.001 | 0% | 0.736 | 2 | 0.99(0.81,1.22) | 0.940 | 26.6% | 0.243 |
| Geographic location |  |  |  |  |  |  |  |  |  |  |
| Europe | 4 | 0.98(0.97,0.99) | 0.003 | 0% | 0.753 | 3 | 0.96(0.93,0.99) | 0.014 | 0% | 0.698 |
| United States | 2 | 0.89(0.82,0.97) | 0.009 | 19.7% | 0.264 | 2 | 1.03(0.80,1.33) | 0.799 | 8.6% | 0.295 |
| Others | 0 | - | - | - | - | 0 | - | - | - | - |
| Sex |  |  |  |  |  |  |  |  |  |  |
| Men | 2 | 0.84(0.76,0.92) | 0.000 | 0% | 0.586 | 1 | 1.28(0.88,1.86) | 0.196 | - | - |
| Women | 3 | 0.93(0.86,1.00) | 0.036 | 52.3% | 0.123 | 2 | 0.89(0.69,1.14) | 0.350 | 0% | 0.786 |
| Size of adenoma |  |  |  |  |  |  |  |  |  |  |
| Small (<10mm) | 3 | 1.01(0.81,1.25) | 0.952 | 28.2% | 0.248 | 2 | 1.08(0.68,1.71) | 0.733 | 0% | 0.699 |
| Large (≥10mm) | 4 | 0.71(0.51,0.99) | 0.042 | 83.7% | 0.000 | 3 | 0.96(0.93,0.99) | 0.014 | 0% | 0.744 |
| No. of cases |  |  |  |  |  |  |  |  |  |  |
| <280 | 1 | 0.98(0.97,0.99) | 0.004 | - | - | 1 | 0.96(0.93,0.99) | 0.016 | - | - |
| ≥280 | 5 | 0.90(0.85,0.95) | 0.000 | 0% | 0.844 | 4 | 0.99(0.83,1.17) | 0.872 | 0% | 0.563 |
| Adjustment for confounders |  |  |  |  |  |  |  |  |  |  |

**Supplemental Table 3.** (Continued)

|  | Yogurt | | | | | Cheese | | | | |
| --- | --- | --- | --- | --- | --- | --- | --- | --- | --- | --- |
| Subgroup | Number  of studies | Pooled RR (95%CI) | *P* value | Heterogeneity | | Number  of studies | Pooled RR (95%CI) | *P* value | Heterogeneity | |
|  |  |  |  | *I^2^* | *P_h_* |  |  |  | *I^2^* | *P_h_* |
| BMI |  |  |  |  |  |  |  |  |  |  |
| Yes | 5 | 0.93(0.87,0.99) | 0.035 | 59.6% | 0.042 | 3 | 0.96(0.93,0.99) | 0.011 | 0% | 0.816 |
| No | 1 | 0.81(0.32,2.06) | 0.658 | - | - | 2 | 1.16(0.87,1.54) | 0.303 | 0% | 1.000 |
| Smoking |  |  |  |  |  |  |  |  |  |  |
| Yes | 5 | 0.93(0.87,0.99) | 0.035 | 59.6% | 0.042 | 3 | 0.96(0.93,0.99) | 0.011 | 0% | 0.816 |
| No | 1 | 0.81(0.32,2.06) | 0.658 | - | - | 2 | 1.16(0.87,1.54) | 0.303 | 0% | 1.000 |
| Alcohol |  |  |  |  |  |  |  |  |  |  |
| Yes | 5 | 0.93(0.87,0.99) | 0.035 | 59.6% | 0.042 | 3 | 0.96(0.93,0.99) | 0.011 | 0% | 0.816 |
| No | 1 | 0.81(0.32,2.06) | 0.658 | - | - | 2 | 1.16(0.87,1.54) | 0.303 | 0% | 1.000 |
| Dietary energy intake |  |  |  |  |  |  |  |  |  |  |
| Yes | 5 | 0.90(0.85,0.95) | 0.000 | 0% | 0.844 | 4 | 0.99(0.83,1.17) | 0.872 | 0% | 0.563 |
| No | 1 | 0.98(0.97,0.99) | 0.004 | - | - | 1 | 0.96(0.93,0.99) | 0.016 | - | - |
| Physical activity |  |  |  |  |  |  |  |  |  |  |
| Yes | 4 | 0.93(0.86,1.00) | 0.043 | 69.1% | 0.021 | 2 | 0.96(0.93,0.99) | 0.640 | 0% | 0.635 |
| No | 2 | 0.83(0.48,1.44) | 0.504 | 0% | 0.951 | 3 | 1.06(0.84,1.33) | 0.013 | 0% | 0.537 |
| Folate |  |  |  |  |  |  |  |  |  |  |
| Yes | 5 | 0.94(0.86,1.02) | 0.002 | 17.1% | 0.306 | 0 | - | - | - | - |
| No | 1 | 0.91(0.86,0.97) | 0.138 | - | - | 5 | 0.96(0.93,0.99) | 0.017 | 0% | 0.711 |

*P_h_:* heterogeneity within each subgroup; NC: not calculable; BMI: body mass index

**Supplemental Table 4.** The sensitivity analysis of the relationship between total milk intake and risk of colorectal adenomas and serrated lesions.

| Excluded studies | No. of included studies | Heterogeneity | | Random-effect model | | |
| --- | --- | --- | --- | --- | --- | --- |
|  |  | *I*^2^ | *P_h_* | RR | 95% CI | *P* |
| **Total milk** | | | |  | | |
| **Case-control studies** | | | |  | | |
| No | 4 | 56.9% | 0.073 | 1.11 | (0.81,1.51) | 0.521 |
| Boutron et al, 1996 | 3 | 71.1% | 0.031 | 1.16 | (0.75,1.79) | 0.493 |
| Kune et al, 1991 | 3 | 0% | 0.798 | 0.96 | (0.79,1.17) | 0.690 |
| Senesse et al, 2002 | 3 | 71.1% | 0.031 | 1.22 | (0.75,1.98) | 0.432 |
| Um et al, 2016 | 3 | 63.0% | 0.067 | 1.26 | (0.80,1.99) | 0.312 |
| **Yogurt** | | | |  | | |
| **All studies** | | | |  | | |
| No | 6 | 50.2% | 0.074 | 0.93 | (0.87,0.99) | 0.029 |
| Kesse et al, 2005 | 5 | 56.8% | 0.055 | 0.93 | (0.87,1.00) | 0.049 |
| Zheng et al, 2019 | 5 | 17.1% | 0.306 | 0.94 | (0.86,1.02) | 0.138 |
| Boutron et al, 1996 | 5 | 59.6% | 0.042 | 0.93 | (0.87,0.99) | 0.035 |
| Karagianni et al, 2010 | 5 | 0% | 0.844 | 0.90 | (0.85,0.95) | 0.000 |
| Rifkin et al, 2020 | 5 | 39.0% | 0.161 | 0.95 | (0.90,1.00) | 0.056 |
| Senesse et al, 2002 | 5 | 59.4% | 0.043 | 0.93 | (0.87,1.00) | 0.036 |

*P_h_:* P value for heterogeneity.
